# Axon–Myelin Interactions during a Viral Infection of the Central Nervous System

**DOI:** 10.1371/journal.ppat.1000519

**Published:** 2009-09-25

**Authors:** Michel Brahic, Jean-Pierre Roussarie

**Affiliations:** 1 Department of Microbiology-Immunology, Stanford University School of Medicine, Stanford, California, United States of America; 2 Molecular and Cellular Neuroscience, The Rockefeller University, New York, New York, United States of America; The Fox Chase Cancer Center, United States of America

Theiler's virus offers a remarkable example of a pathogen that navigates the various cells of the organism to evade immune responses and establish a persistent infection. Here, we discuss the transition from neuron to myelin and oligodendrocyte infection, a step that is crucial for the persistence of this virus in the central nervous system (CNS).

CNS myelin is an extension of the cytoplasmic membrane of oligodendrocytes wrapped numerous times around axons. An oligodendrocyte sends many such extensions and can myelinate up to 50 different axons. Myelinated axon segments are separated by short unmyelinated regions called nodes of Ranvier. Cytoplasm is totally extruded from myelin except in areas where it forms channels that are in continuity with the oligodendrocyte cell body. These channels form the so-called ad-axonal inner loop and the paranodal loops at the level of the nodes of Ranvier. Inner and paranodal loops are in close contact with the axon membrane (Figure 1). (For a review of myelin and node organization, see [1].)

**Figure 1:**
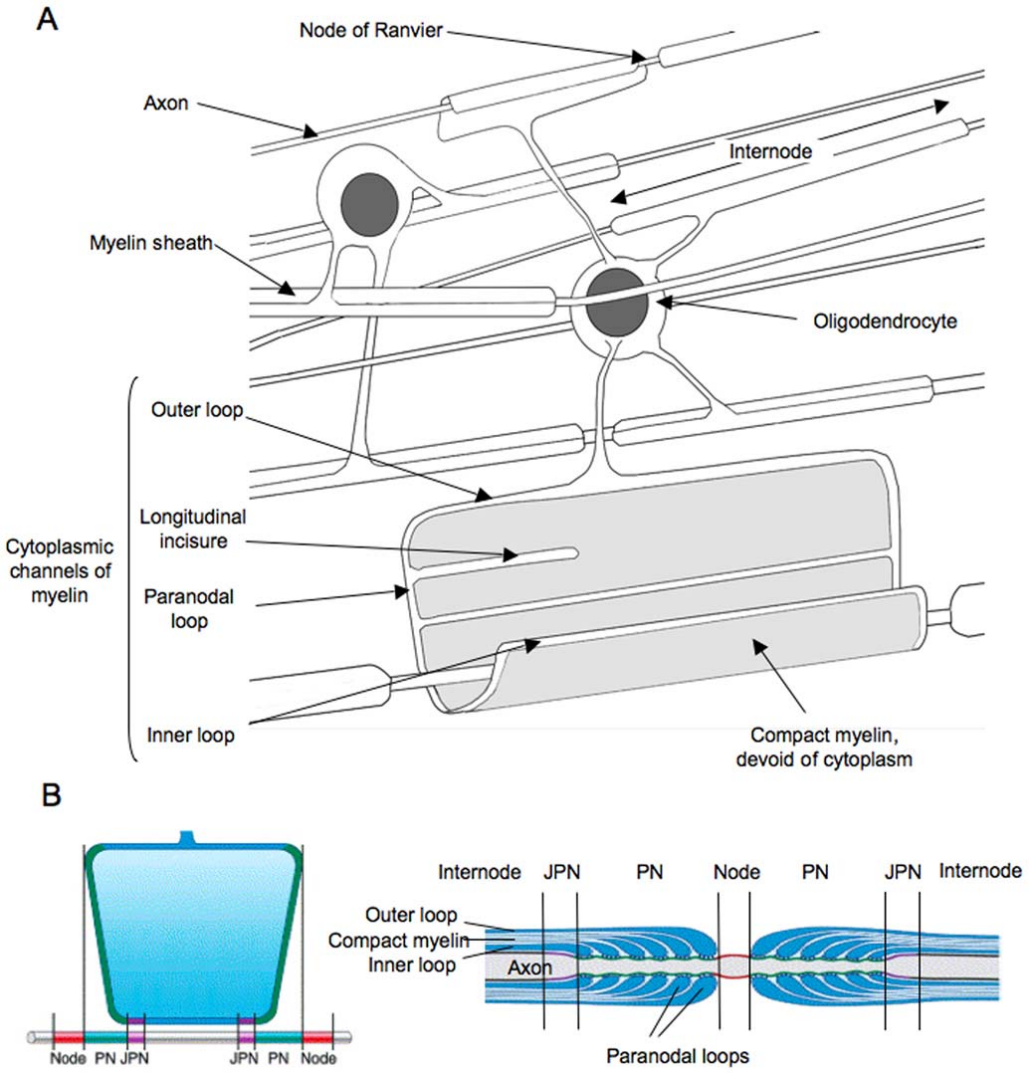
Diagrammatic view of CNS myelin. Myelin is an extension of the plasma membrane of the oligodendrocyte. Compact myelin is devoid of cytoplasm. Cytoplasmic channels, which are continuous with the oligodendrocyte cytoplasm, form the so-called inner and outer myelin loops as well as the longitudinal incisures.

## Theiler's Virus

Theiler's virus, a mouse picornavirus, is responsible for a peculiar neurological disease. It infects neurons and spreads by fast axonal transport for approximately 2 weeks following intracerebral inoculation. Depending on their genetic background, mice may clear this infection or remain persistently infected. The virus is no longer in neurons during persistent infection. Instead, it is found in white matter, in oligodendrocytes, in the cytoplasmic channels of myelin, and in macrophages. The infection is focal and results in inflammation, primary demyelination, and some axonal damage. For more detailed reviews of Theiler's virus and its pathogenesis, the reader is referred to [2],[3].

In the course of studying this persistent infection, our laboratory observed that two myelin mutants, the *shiverer* and *rumpshaker* mice, were totally resistant to persistent infection, whereas the parental strains were susceptible [4]. These strains bear mutations in the *Mbp* and *Plp1* genes, which encode two major myelin components, myelin basic protein (MBP) and proteolipid protein (PLP), respectively. *Shiverer* mice make very little myelin, whereas in *rumpshaker* mice, the amount of myelin is reduced and the periodicity of myelin leaflets is slightly altered [5],[6]. Theiler's virus infects neurons and is transported in axons in these mutant mice just as in wild-type mice. However, it disappears from the CNS of the mutants after 2–3 weeks. Various experiments showed that this clearance was not immune mediated and that *shiverer* oligodendrocytes grown in culture were permissive to viral replication [7]. These results suggested that myelin might play a crucial role in viral persistence. What could that role be?

We postulated that viruses transported in axons infect the surrounding myelin and spread to oligodendrocyte cell bodies, then to macrophages, where infection persists. Accordingly, myelin would be an obligatory passage that would be prevented by the *shiverer* and *rumpshaker* mutations. We tested this hypothesis by introducing virus in the vitreous chamber of the eye. The virus infected retinal ganglion cells and was transported anterogradely in the axons of the optic nerve. Infected oligodendrocytes were observed in the nerve of wild-type mice as early as 3 days post-inoculation. The only possible source of infection for these oligodendrocytes is the axons of retinal ganglion cells. In contrast, the infection of oligodendrocyte cell bodies was considerably impaired in *shiverer* and *rumpshaker* mice, although the number of oligodendrocytes is normal in these mutants, supporting our hypothesis [7]. Importantly, by performing these experiments with *Wld^s^* mice, a mutant whose axons are strikingly resistant to degeneration, we showed that the virus was able to traffic from axon to myelin in the absence of axonal degeneration [8].

Enveloped viruses with their lipid bilayer and glycoproteins can exit the cytoplasm by budding from the plasma membrane and entering a new host cell by fusion. Classically non-enveloped viruses such as picornaviruses exit by causing cell lysis. How can Theiler's virus cross from intact axons into the surrounding myelin? Before presenting hypotheses it is necessary to review briefly the salient aspects of axon–myelin interactions.

## Axon–Myelin Interactions

Mice and humans with myelin mutations have provided most of our understanding of axon–myelin interactions. PLP is a major myelin protein. *Plp1nul* mice have myelinated axons, surprisingly, and mice are normal at birth [9]. However, in late life, organelles accumulate in the axons at the level of the nodes of Ranvier and there is axonal swelling and degeneration [10]. The *Plp1* gene is on the X chromosome. Because in females one of the X chromosomes is inactivated, the oligodendrocytes of a female heterozygous for a *Plp1* mutation form a mosaic of wild-type and mutant cells. In these mice, the same axon can be myelinated alternatively by wild-type and by mutant oligodendrocytes. Remarkably, axonal swelling is observed only in axon segments myelinated by mutant oligodendrocytes [11]. These elegant experiments show that a myelin protein, PLP, has a direct role in maintaining the integrity of the axon.

In the CNS, 2′,3′ cyclic nucleotide 3′-phosphodiesterase (CNPase) is found only in oligodendrocyte cell bodies and in myelin [12]–[14]. *Cnp1−/−* mice have normal myelin. However, with age, they develop a neurological deficit due to abnormal distribution of ion channels in the nodes of Ranvier [15]. The CNPase mutation nicely segregates two functions of the oligodendrocyte: the production of myelin, which is unaffected, and the support of the axon, which requires the presence of the enzyme.

In contrast to these mutant phenotypes, and others which we did not discuss, there is no axonal degeneration in the *shiverer* mouse even though the amount of myelin is severely restricted, indicating that myelin supports the axon through specific signaling and not just by the physical presence of an electric insulator [10].

Lastly, axon–myelin interactions may include cytoplasmic exchanges between the two compartments. The giant axons of invertebrates, such as squids and crayfish, are surrounded by uncompacted multilamellar glial sheaths whose main function appears to be axonal support more than electric insulation. The exchange of macromolecules between the axon and this periaxonal glia is well documented [16]. It may take place through cytoplasmic channels that connect both compartments as well as by an exchange of vesicles budding from one compartment and fusing with the other one [17],[18]. Therefore, axonal support appears to be an ancestral function of glial cells that predates electric insulation. Cytosol exchanges between axon and myelin may still take place in vertebrates. In peripheral nerves, myelin forms complex multilamellar plasma membrane structures, called axon-Schwann cell networks, that invade the axon. They become prominent following distal axon injury and appear to engulf axon material such as neurofilaments, microtubules, and mitochondria. The reader is referred to the article by Spencer and Thomas [19] and to the electron micrographs published by Gatzinsky et al. [20] for more information on this subject. Axon-Schwann cell networks could be important for clearing retrogradely transported organelles targeted for degradation, thereby relieving the neuron cell body from the burden of recycling the large volume of cytoplasm present in long axons and preventing toxic products introduced into peripheral axons from reaching the cell body [21]–[24].

Structures analogous to the axon-Schwann cell networks have been described by electron microscopy in CNS myelin [19],[22],[25]. They may function for axon clearance, as indicated by several observations. For example, Lucifer yellow and horseradish peroxidase injected into the eye of mice can be found in the myelin of the optic nerve [26],[27]. In a transgenic mouse model of Huntington disease, huntingtin aggregates formed in neurons are found in the myelin surrounding degenerating axons [28]. Multiple system atrophy is a rare human disease characterized by the presence of alpha-synuclein inclusions in oligodendrocytes [29]. Since alpha-synuclein is not normally expressed in oligodendrocytes, and since alpha-synuclein mRNA is not detected in oligodendrocytes containing alpha-synuclein inclusions, the protein is most likely imported from the axons into the oligodendrocyte [30].

In summary, the role of myelin is much more complex than that of a passive electric insulator. Through signaling pathways that are still largely unknown, myelin is one of the factors that determines the cytoarchitecture of the axon and provides axons with support that, if abolished, leads to axonal degeneration. The myelin of Schwann cells and possibly that of oligodendrocytes may also be important in clearing the axon of unwanted organelles and insoluble protein aggregates.

## Theiler's Virus Traffic within the CNS

**Figure 2:**
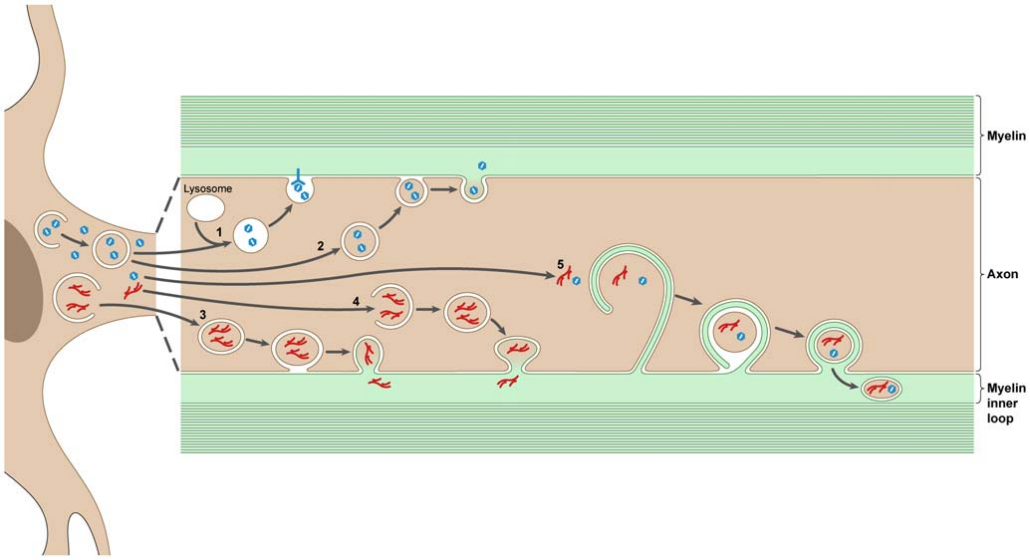
Hypothetical mechanisms for the traffic of Theiler's virus from the axon into the surrounding myelin, in the absence of axonal degeneration. Viral particles are shown in blue, replication complexes in red. Pathway 1: Viral particles (blue) are engulfed in double-membrane autophagosomes. Following fusion of the autophagosome with a lysosome and digestion of its inner membrane, the particles, which are resistant to low pH and to proteases, are in a single-membrane vesicle that fuses with the axolemma, thereby releasing the virus in the periaxonal space. Entry in the myelin requires the presence of a viral receptor. Pathway 2: The outer membrane of the double-membrane vesicle fuses with the axolemma. The single-membrane vesicle that is released from the axon fuses with the membrane of the myelin inner loop and delivers viral particles into the myelin. This is an unlikely pathway since the viral RNA cannot be released from the virus particle without interaction with a receptor. Pathway 3: A pathway similar to pathway 2, but in this case replication complexes (red), instead of viral particles, are transferred from the axon into the myelin where replication can resume. Pathway 4: Engulfment of replication complexes may take place in the axon, where autophagy is known to be prominent. Pathway 5: Viral particles or replication complexes are transferred from the axon into the myelin by a hypothetical mechanism analogous to the axon clearing mechanism described in peripheral nerves [21]. A double membrane (axolemma plus myelin) engulfs axonal material, including viral products. Two fusion events, (axolemma/axolemma) and (myelin/myelin), result in the intoduction of a double-membrane vesicle containing viral material into the myelin inner loop.

We will outline a few hypothetical mechanisms by which Theiler's virus could traffic from intact axons into myelin (see Figure 2 and its legend). They are based on the fact that viruses, in particular those with limited genetic information, tend to hijack cellular functions for their replication and spread.

First, it has been proposed that picornaviruses could exit through an intact plasma membrane by a mechanism derived from autophagy. Cellular autophagy consists of the engulfment of cytoplasmic material in a double-membrane vesicle that fuses with endosomes and then lysosomes, causing the inner membrane and its contents to be degraded [31]. In an infected cell microbes may be engulfed in the double-membrane vesicle. Therefore, autophagy can be considered part of the innate immune response. However, picornaviruses are highly resistant to proteases and low pH and may resist degradation. If the single-membrane vesicle were to fuse with the plasma membrane, it would deliver viral particles to the outside in the absence of cell lysis [32] (Figure 2, pathway 1). Interestingly, autophagy is very active in neurons, including in axons. In a variation on this theme, double-membrane vesicles could engulf viral RNA replication/translation complexes. They could then fuse with the plasma membrane instead of with a lysosome, releasing a single-membrane vesicle into the extracellular milieu. Such a vesicle could fuse with the plasma membrane of a neighboring cell and deliver replication complexes directly into the cytoplasm where they could resume their activity (K. Kirkegaard, personal communication) (Figure 2, pathways 3 and 4).

Second, we discussed above how CNS myelin may play a role in clearing the axon of unwanted materials, in particular organelles targeted for degradation. We summarized the evidence suggesting that such clearance involves the transport of axonal cytosol into the internal and paranodal loops of myelin. Theiler's virus could have found ways to introduce itself into such a pathway, thereby gaining access to the cytoplasmic channels of myelin. In such a scenario, the viral material transferred would be replication/translation complexes, not virions. Indeed, virions introduced directly into the cytoplasm are not infectious because decapsidation requires interaction with the viral receptor. Viral replication/translation complexes, on the other hand, could resume their activity upon entry into myelin (Figure 2, pathway 5).

## Conclusions

Microorganisms, and viruses in particular, are masters at using the peculiarities of the organ which they infect. For this reason, they can often be used as probes to uncover new, unsuspected, physiological mechanisms. Therefore, pathogenesis is as much a study of the normal functioning of the organ as it is a study of the pathogen. Intercellular communication, which is essential for all multicellular organisms, is paramount to the functioning of the nervous system. Communication between neurons at the level of synapses has been known as the basis of CNS functioning for a long time. Communication between glial cells and between glial cells and neurons has been appreciated only more recently. Dissecting, at the molecular level, the mechanism by which Theiler's virus crosses from the axon into the myelin, a step required for its persistence in CNS, will help fill a gap in this important chapter of neuroscience.
